# The Association between Early-Onset Pancreatic Ductal Adenocarcinoma and Patients Survival: A Systematic Review and Meta-Analysis

**DOI:** 10.12688/f1000research.153743.1

**Published:** 2024-08-28

**Authors:** Kaka Renaldi, Andy William

**Affiliations:** 1 Division of Gastroenterology, Department of Internal Medicine, Faculty of Medicine University of Indonesia/Cipto Mangunkusumo National General Hospital, Central Jakarta, Jakarta, 10430, Indonesia; 2 Faculty of Medicine, University of Indonesia, Jakarta, Indonesia

**Keywords:** Meta-analysis, Pancreatic neoplasms, Survival, Young adult

## Abstract

**Background:**

In recent years, the incidence of early-onset pancreatic cancer (EOPC) has increased. Several studies comparing the survival of patients with EOPC to those with average-onset pancreatic cancer (AOPC) have reported mixed results. We aimed, therefore, to perform a meta-analysis summarizing the current evidence.

**Methods:**

We searched the MEDLINE and EMBASE databases for relevant articles published through March 2024. Articles comparing the survival of patients with EOPC – defined as pancreatic ductal adenocarcinoma (PDAC) diagnosed at ≤ 50 years of age – and AOPC were included in the present meta-analysis. The primary outcome was the pooled adjusted hazard ratio (aHR), and the risk of bias analysis was performed using the Quality in Prognostic Factor Studies tool. The meta-analysis was performed using a random-effects model.

**Results:**

A total of 17 studies were eligible for the primary analysis, the results of which indicated that patients with EOPC had a longer overall survival than those with AOPC (aHR = 0.80; 95% confidence interval [CI], 0.74–0.86; *P* < 0.001). The rate of distant metastasis was higher in EOPC than AOPC; however, patients with EOPC also received more treatments than those with AOPC.

**Conclusions:**

Patients with EOPC had a better prognosis than those with AOPC. Clinicians must ensure that patients with PDAC receive early and appropriate treatment to improve their survival.

## Introduction

As of 2022, pancreatic cancer is the 12 ^th^ most common type of cancer and the 6 ^th^ largest contributor of cancer-related mortality worldwide, with a relative 5-year survival rate of 12.5%. ^1^ According to the Surveillance, Epidemiology, and End Results (SEER) database, the median age of patients diagnosed with pancreatic cancer is 70 years old ^2^; however, the number of younger patients diagnosed with pancreatic cancer, termed early-onset pancreatic cancer (EOPC), is increasing. ^3^

There is no consensus regarding the definition of EOPC as it pertains to age; however, it is largely used to describe patients who are ≤ 50 years of age when diagnosed with pancreatic cancer. ^4^^–^^7^ Additionally, pancreatic cancer that is diagnosed at age < 45 years old is occasionally defined as very early-onset pancreatic cancer (VEOPC). ^8^ EOPC accounts for 0.87–11.50% of pancreatic cancers, depending on the study population. ^9^^,^^10^ Although EOPC occurs more often in males, the age-adjusted incidence rate increased significantly more in young females than their male counterparts. ^11^ Several risk factors have been associated with EOPC, including heavy alcohol consumption, smoking, family history of pancreatic cancer, diabetes mellitus, obesity, and pancreatitis. ^12^ Some studies also showed unique molecular profiles in patients with EOPC, such as a higher frequency of wild-type *KRAS* and higher mutation rates of *CDKN2A*, *SMAD4*, and *FOXC2.* ^13^ Although the effects of these genomic alterations on tumor behavior are still unclear, some studies have shown that patients with EOPC often present with higher rates of distant metastasis. ^14^

Studies investigating differences in survival times between patients with early-onset PDAC and those with average-onset PDAC (AOPC) have shown conflicting results. Several studies have shown that patients with EOPC have a better prognosis than those with AOPC ^4^^,^^15^; however, some studies have shown that patients with EOPC have a worse survival than those with AOPC. ^9^^,^^14^ Additionally, some studies did not find any significant difference in survival between patients with EOPC and those with AOPC. ^5^^,^^7^ To the best of the authors’ knowledge, however, a meta-analysis on this topic has not yet been performed. In the present study, therefore, we conducted a meta-analysis of studies that compared the survival rates of patients with EOPC to those with AOPC, specifically focusing on pancreatic ductal adenocarcinoma (PDAC), which is the most common type of pancreatic cancer (approximately 90%). ^16^

## Methods

The present systematic review and meta-analysis was conducted following the Preferred Reporting Items for Systematic Reviews and Meta-analyses (PRISMA) checklist. ^17^

### Inclusion and exclusion criteria

We used the PICOTS framework ^18^ to define the review questions, as follows: *Population* = patients with PDAC; *Index Prognostic Factor* = EOPC; *Comparator Prognostic Factor* = adjusted for cancer stage/resectability status/tumor size; *Outcome* = Survival; *Timing* = age at diagnosis; *Setting* = all care settings. Based on the review question, the inclusion criteria were as follows: 1) studies that compared the overall survival between patients with EOPC and those with AOPC, although to maximize the number of studies included, if only cancer-specific survival were available, studies were still accepted; 2) studies that defined EOPC as patients who were diagnosed with PDAC at ≤ 50 years of age (although various definitions of EOPC are used in the relevant literature, we chose this cut-off because it was the most frequently used definition in the literature ^4^^–^^7^^,^^9^^,^^14^^,^^19^^–^^24^; by this definition, studies that used a cut-off of 45 or 40 years of age were also included, and the comparator group in each study was categorized as the AOPC group); and 3) survival analysis must be adjusted to the tumor stage (either by regression analysis or matching) – if the American Joint Committee on Cancer (AJCC) cancer stage ^25^ was unavailable, we also accepted survival analysis, which was adjusted for tumor resectability or tumor size to maximize the number of studies included. The exclusion criteria were as follows: 1) studies with only abstracts available; 2) studies that did not present a hazard ratio and were inestimable from other values by the methods described by Tierney et al. ^26^ and Hebert et al. ^27^; and 3) studies that were not available in English.

### Search strategy

Two independent reviewers searched the MEDLINE and EMBASE databases for articles published through March 2024. For MEDLINE, the following search terms were used: (early-onset pancreatic cancer [Title/Abstract]) OR ((young [Title/Abstract] AND pancreatic cancer [Title/Abstract])). For EMBASE, the following terms were used: ‘early-onset pancreatic cancer’:ab,ti OR (young:ab,ti AND ‘pancreatic cancer’:ab,ti). We also reviewed the reference lists of related papers to identify additional studies.

### Data extraction

Two independent reviewers extracted the data from included studies using the Checklist for Critical Appraisal and Data Extraction for Systematic Reviews of Prognostic Factor Studies (CHARMS-PF). ^18^ The following data were extracted: authors; year of study; study design; definition of EOPC and AOPC; number of subjects; period of recruitment; evaluation of survival; median follow-up; adjustment to other variables; hazard ratio; rate of distant metastasis; and proportion of subjects who received surgery, chemotherapy, or radiotherapy. Additionally, the risk of bias for each study was analyzed using the Quality in Prognostic Factor Studies (QUIPS) tool, ^28^ which evaluates the following aspects: adequate study participation; study attrition; prognostic factor measurement; outcome measurement; adjustment for other prognostic factors; and statistical analysis and reporting. Permission has been obtained from the creator to use the QUIPS tool.

### Statistical analysis

The Review Manager (RevMan) 5.3 program (The Nordic Cochrane Center, Copenhagen, Denmark) ^29^ and R 4.3.2 program (R Core Team, Vienna, Austria) ^30^ were used to perform the meta-analysis. The primary outcome was overall survival. The adjusted hazard ratio (aHR) for each study was used in the pooled analysis and presented as forest plots. If there was a study in which survival analysis was stratified based on cancer stage or resectability, multiple hazard ratios were first pooled into one value. If the aHR was not stated, it was estimated using the methods described by Tierney et al. ^26^ and Hebert et al. ^27^ If there was substantial heterogeneity between the studies, we used a random effects analysis to calculate the pooled aHR, otherwise, a fixed-effects analysis was used. Additionally, we performed a pooled hazard ratio analysis for cancer-specific survival (CSS), disease-free survival (DFS), progression-free survival (PFS), and recurrence-free survival (RFS).

We also performed sensitivity analyses, based on the type of survival analysis, age cutoff, presence of matching, regression analysis, published hazard ratio only, and adjustment for several covariates, as well as a subgroup analysis of the patients who underwent surgery. Publication bias was assessed using funnel plots, and Egger’s test was performed using ProMeta 3 (Internovi, Cesena, Italy). ^31^ Additionally, we calculated the pooled risk ratios for the rates of distant metastasis (stage IV cancer) and treatment (surgery, chemotherapy, and radiotherapy).

## Results

### Study selection

**Figure 1.**
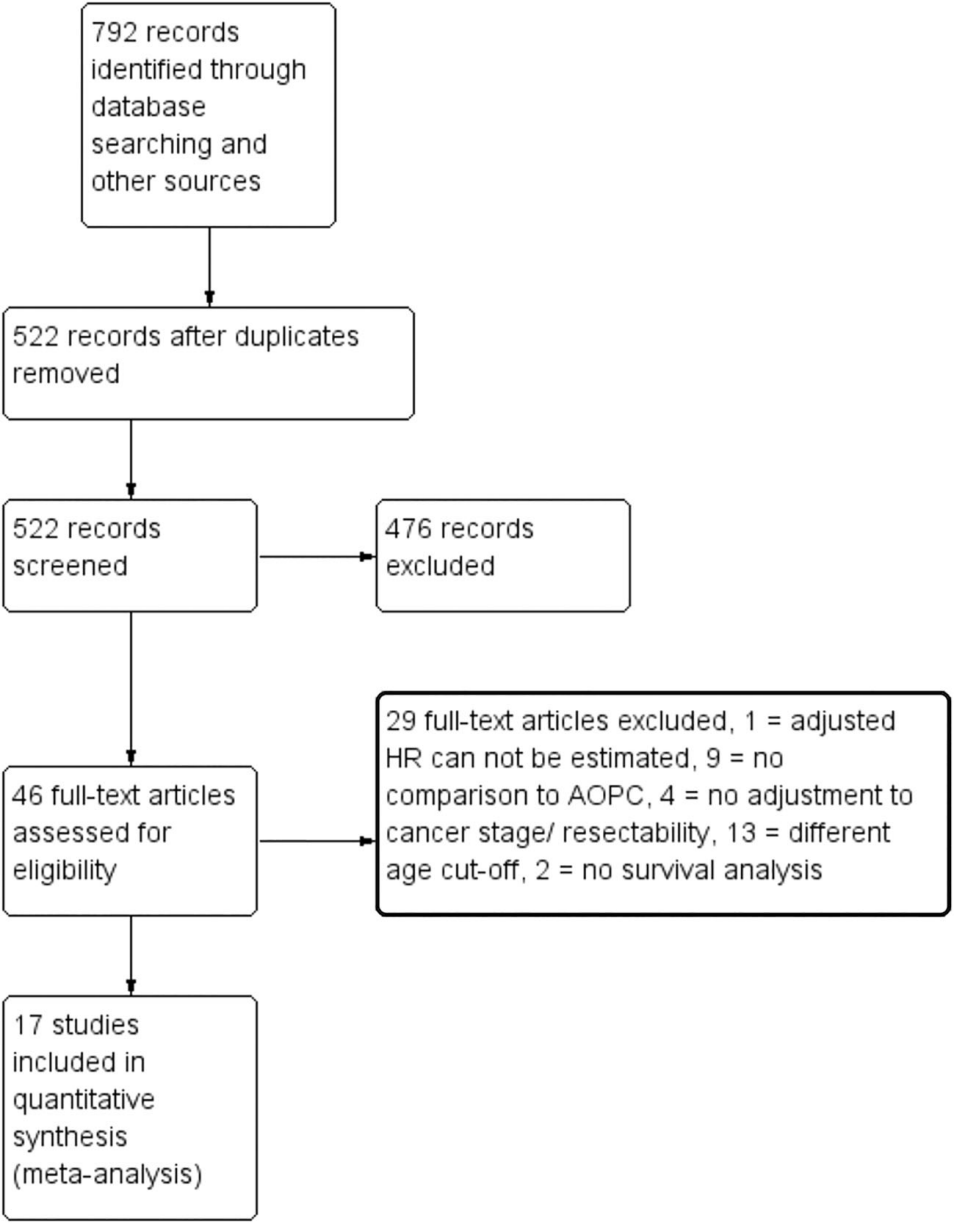
PRISMA study flow diagram.

The PRISMA study flow diagram is shown in Figure 1. A total of 522 records were initially obtained from MEDLINE, EMBASE, and the reference lists of the eligible studies, after the removal of duplicates, of which 46 were assessed for eligibility. Articles that only included patients with EOPC or used different age cutoffs for EOPC (e.g., age < 55, < 60, or < 70 years) were excluded. Table S1 shows a list of the excluded studies and the reasons for their exclusion. In total, 17 studies were included in the final analysis.

### Study characteristics

Table 1 shows the characteristics of the 17 studies included in the present meta-analysis. The total number of patients with EOPC was 33,274 subjects, while that of those with AOPC was 563,198.

**Table 1.** Summary of included studies.

No.	Study	Definitions	% of EOPC patients in population	No. of EOPC patients	No. of AOPC patients	Place & period or recruitment	Median follow-up	Adjustment to survival analysis	Adjusted HR (EOPC vs. AOPC)	Median survival & survival rates (EOPC vs. AOPC)
1.	Kang JS 2017	EOPC = <45 y.o.; AOPC = 45 y.o.	4.90%	- 34 (total, before PSM) - 34 (after PSM)	- 665 (total, before PSM) - 68 (after PSM)	South Korea, 2000–2014	Not stated	No Cox regression, only PSM. PSM adjusted with patients' ASA score, AJCC stage, adjuvant chemotherapy and radiotherapy.	Not stated	- Median OS: 17 months vs. 32 months; P = 0.970 - 5-year OS: 5.4% vs. 18.0%
2.	Ansari D 2019	EOPC = <50 y.o.; LOPC = ≥ 50 y.o.	6.20%	- 3172 (after PSM)	- 3172 (after PSM)	USA, 2004–2016	Not stated	Gender, tumor size, AJCC stage 7th edition, surgery, and chemotherapy	aHR = 1.07 (95% CI 1.01–1.13; P = 0.015)	- Median survival: Not stated - 5-year OS: 6.1% vs 8.6%, p = 0.003 - 5-year CSS: 6.7% vs 9.7%, p < 0.001
3.	Saadat LV 2021	EOPC = <50 y.o.; AOPC = >= 50 y.o.	6.30%	11161 (treated pts)	92387 (treated pts)	USA, 2004–2016	30.2 months	No Cox regression, although there were subgroup analyses based on treated and untreated patients, stage 0–2 disease, stage 3–4 disease, and time period	Not stated	- Median survival: Not stated - 1-year OS for stage 0-2 disease: 72.4% (95%CI: 71.2%–73.7%) vs. 53.3% (95%CI: 52.9%–53.7%) - 1-year OS for stage 3 disease: 47.6 (95%CI: 45.1%–50.0%) vs 37.8% (95%CI: 37.1%–38.4%) - 1-year OS for stage 4 disease: 24.8% (95%CI: 23.8%–25.8%) vs 14.8% (95%CI: 14.5%–14.9%)
4.	Dai D 2019	EOPC = < 45 y.o. vs. older group	2.50%	1386	53932	USA, 2004–?	Not stated	Age, sex, race, tumor location, surgery experience, tumor size, lymph node ratio, 6 ^th^ AJCC TNM stage, grade, radiotherapy & chemotherapy experience, marital status	- aHR age < 45 y.o. vs. 45–59 y.o. = 0.93 (95% CI 0.88–0.98; P = 0.010) - aHR age < 45 y.o. vs. 60–69 y.o. = 0.91 (95% CI 0.85–0.96; P = < 0.001) - aHR age < 45 y.o. vs. 70–79 y.o. = 0.86 (95% CI 0.81–0.92; P = < 0.001) - aHR age < 45 y.o. vs. >79 y.o. = 0.85 (95% CI 0.81–0.91; P = < 0.001)	Not stated
6.	Piciucchi M 2015	EOPC = ≤ 50 y.o. at diagnosis, AOPC = age > 50 y.o. at diagnosis	8.50%	25	268	Italy, 2006–2013	Not stated	Tumor stage included, others unclear	aHR = 0.7; (95% CI 0.4–1.1; p = 0.1)	- Median OS = 11 months vs. 9 months; P = 0.28
6.	Tingstedt B 2011	EOPC = ≤ 50 y.o. at diagnosis, AOPC = age > 50 y.o. at diagnosis	5.70%	33	33	Sweden, Jan 1993–Dec 2008	Not stated	No Cox regression, but patients were matched with controls based on sex, resection, tumor size, chemotherapy and radiotherapy	Not stated	- Median OS: 5.67 months vs. 8.00 months; P = 0.12 - 5-year OS: 3.3% vs. 0%
7.	He J 2013	EOPC = ≤ 45 y.o., LOPC = ≥ 70 y.o.	7.90%	75	870	USA, 1975–2009	Not stated	No Cox regression, only subgroup analysis based on cancer stage	Not stated	- Median OS: 19 months vs. 16 months; P = 0.007 - 5-year OS = 24% vs. 11%; P = 0.005 - 10-year OS = 17% vs. 3%, P < 0.001
8.	Ordonez JE 2020	EOPC = <50 y.o.; AOPC = ≥ 50 y.o. at diagnosis	5.90%	12137	194925	USA, 2004–2013	Not stated	Age, sex, race/ethnicity, comorbidities, insurance status, tumor size, anatomic location, tumor grade/differentiation, lymph node status, AJCC stage, presence of lymphovascular invasion, and receipt of surgery, chemotherapy, or radiation	0.867 (95% CI 0.85–0.88)	- Median OS: 9.2 months vs. 6.0 months; P < 0.001
9.	Beeghly-Fadiel A 2016	EOPC = <50 y.o.; AOPC = ≥50 y.o. at diagnosis	11.50%	118	1282	USA, 1988─2013	Not stated	Age, race, year of diagnosis, AJCC stage, tumor location, treatment received, multiple malignancies, family history of pancreatic cancer	0.82 (95% CI 0.67─1.00)	- - Median OS: 9.36 months vs. 8.04 months; P = 0.403
10.	Whitley A 2023	EOPC = ≤ 50 y.o. at diagnosis, AOPC = age > 50 y.o. at diagnosis	7.00%	1324	17564	Czech Republic, 1985─2015	Not stated	No Cox regression, but had subgroup analysis based on the stage of cancer	Not stated	- Median OS: 5.9 months vs. 4.5 months; P < 0.001 - 1-year OS: 28.4% vs. 22.6%; P < 0.001 - 2-year OS: 15.3% vs. 10.1%; P < 0.001 - 3-year OS: 11.4% vs. 6.6%; P < 0.001 - 5-year OS: 8.2% vs. 4.0%; P < 0.001
11.	Castet F 2023	EOPC = ≤ 50 y.o. at diagnosis, AOPC = age ≥ 70 y.o. at diagnosis	Not stated	139	141	Spain, 2010─2022	54.8 months	Sex, history of diabetes, tobacco history, alcohol intake, clinical stage, tumor location, ECOG performance status (ECOG-PS), CA19.9 levels, albumin levels, and neutrophil-to-lymphocyte ratio (NLR)	0.87 (95% CI 0.65─1.16; P = 0.33)	- Median OS: 18.7 months vs. 17.6 months; P = 0.75
12.	Zironda A 2023	EOPC = ≤ 50 y.o. at diagnosis, AOPC = age > 50 y.o. at diagnosis	5.70%	65	1068	USA, Jan 2011─Dec 2021	22.4 months	Onset of PDAC, age, race, sex, ASA, diabetes, elevated Ca 19-9, neoadjuvant therapy, adjuvant therapy, minimally invasive surgery approach, vascular resection, major complication, IPMN pathology, tumor size, grade, lymph node involvement, R0 resection	0.93 (95% CI 0.64─1.33; P = 0.68)	- Median OS: 30.6 months vs. 31.0 months - 1-year OS: 73.3% vs. 79.5% - 3-year OS: 43.9% vs. 43.9% - 5-year OS: 33.0% vs. 31.0%
13.	Takeda T 2022	EOPC = ≤ 50 y.o. at diagnosis, AOPC = age > 50 y.o. at diagnosis	8.00%	127	1519	Japan, Jan 2010─Aug 2019	Not stated.	No Cox regression, but had subgroup analysis based on resectability of cancer	Not stated	- Median OS: 16.9 months vs. 17.1 months; P = 0.565
14.	Ren S 2023	EOPC = < 50 y.o. at diagnosis, AOPC = age ≥ 50 y.o. at diagnosis	6.90%	763	2278	USA, 2004─2018	Not stated	Sex, race, site, tumor differentiation, TNM stage and treatment patterns	Not stated	- Median OS: 9 months vs. 8 months; P = 0.002 - 1-year OS: 38.4% vs. 36.8% - 3-year OS: 11.1% vs. 10.1% - 5-year OS: 6.9% vs. 5.8%
15.	Ramai D 2021	EOPC = ≤ 40 y.o. at diagnosis, AOPC = age > 40 y.o. at diagnosis	0.87%	1181	134919	USA, 1975─2016	Not stated	Age, sex, race, tumor grade, stage, T status, N status, primary tumor site, no. of lymph node examined, no. of positive lymph nodes, receipt of surgery, chemotherapy, or radiation	0.485 (95% CI 0.422–0.557, P < 0.001)	- Median OS: 7.0 months vs. 6.0 months; P < 0.001
16.	Wang H 2020	EOPC = ≤ 40 y.o. at diagnosis, AOPC = age > 40 y.o. at diagnosis	1.12%	1422	57201	USA, 2004─2015	Not stated	Race, gender, year of diagnosis, pathological grade, AJCC stage, historic stage, tumor location	- aHR age 20–40 vs. 40–60 = 0.54 (95% CI 0.50–0.58; P < 0.001) - aHR age 20–40 vs. 60–80 = 0.45 (95% CI 0.42–0.49; P < 0.001) - aHR age 20–40 vs. >80 = 0.30 (95% CI 0.28–0.33; P < 0.001)	- Median CSS age 20–40 vs. age 40–60 vs. age 60–80 vs. age >80 = 36.0 months vs. 10.0 months vs. 8.0 months vs. 4.0 months - 5-year CSS age 20–40 vs. age 40–60 vs. age 60–80 vs. age >80: 44.7% vs. 16.9% vs. 13.8% vs. 8.7%
17.	Mendis S 2024	EOPC = ≤ 50 y.o. at diagnosis, AOPC = age > 50 y.o. at diagnosis	6.70%	112	1571	Australia, New Zealand, Singapore, Jan 2016–Dec 2021	23.6 months	No Cox regression, but has subgroup analysis based on tumor resectability	- aHR locally advanced = 0.47 (95% Cl 0.32–0.69; P = 0.005) - aHR metastatic = 0.66 (95% Cl 0.48–0.89; P =0.025)	- Median OS: 23.4 months vs 10.3 months - P < 0.001

Abbreviations: EOPC = early-onset pancreatic cancer; AOPC = average-onset pancreatic cancer; aHR = adjusted hazard ratio; AJCC = American Joint Committee on Cancer, ECOG-PS = Eastern Cooperative Oncology Group - Performance Status; OS = overall survival; PSM = propensity score matching; CSS = cancer-specific survival; IPMN = intraductal papillary mucinous neoplasms; y.o. = years old.

### Risk of bias

Table 2 shows the risk of bias for the 17 studies included in the present meta-analysis.

**Table 2.** Risk of bias of included studies.

No.	Study	Study participation	Study attrition	Prognostic factor measurement	Outcome measurement	Adjustment for other prognostic factors	Statistical analysis & reporting
1.	Kang JS 2017	Moderate	Moderate	Low	Low	Low	Moderate
2.	Ansari D 2019	Low	Low	Low	Low	Low	Low
3.	Saadat LV 2021	Moderate	Low	Low	Low	Moderate	Moderate
4.	Dai D 2019	Low	Low	Low	Low	Low	Low
5.	Piciucchi M 2015	Moderate	Moderate	Low	Low	Moderate	Low
6.	Tingstedt B 2011	Low	Moderate	Low	Low	Low	Moderate
7.	He J 2013	Low	Low	Low	Low	Moderate	Moderate
8.	Ordonez JE 2020	Low	Low	Low	Low	Low	Low
9.	Beeghly-Fadiel A 2016	Low	Low	Low	Low	Low	Low
10.	Whitley A 2023	Low	Low	Low	Low	Moderate	Moderate
11.	Castet F 2023	Low	Low	Low	Low	Low	Low
12.	Zironda A 2023	Low	Low	Low	Low	Low	Low
13.	Takeda T 2022	Low	Low	Low	Low	Moderate	Moderate
14.	Ren S 2023	Low	Low	Low	Low	Low	Moderate
15.	Ramai D 2021	Moderate	Low	Low	Low	Low	Moderate
16.	Wang H 2020	Low	Low	Low	Low	Low	Low
17.	Mendis S 2024	Low	Low	Low	Low	Moderate	Low

### Meta-analysis

**Overall survival (OS)**

**Figure 2.**
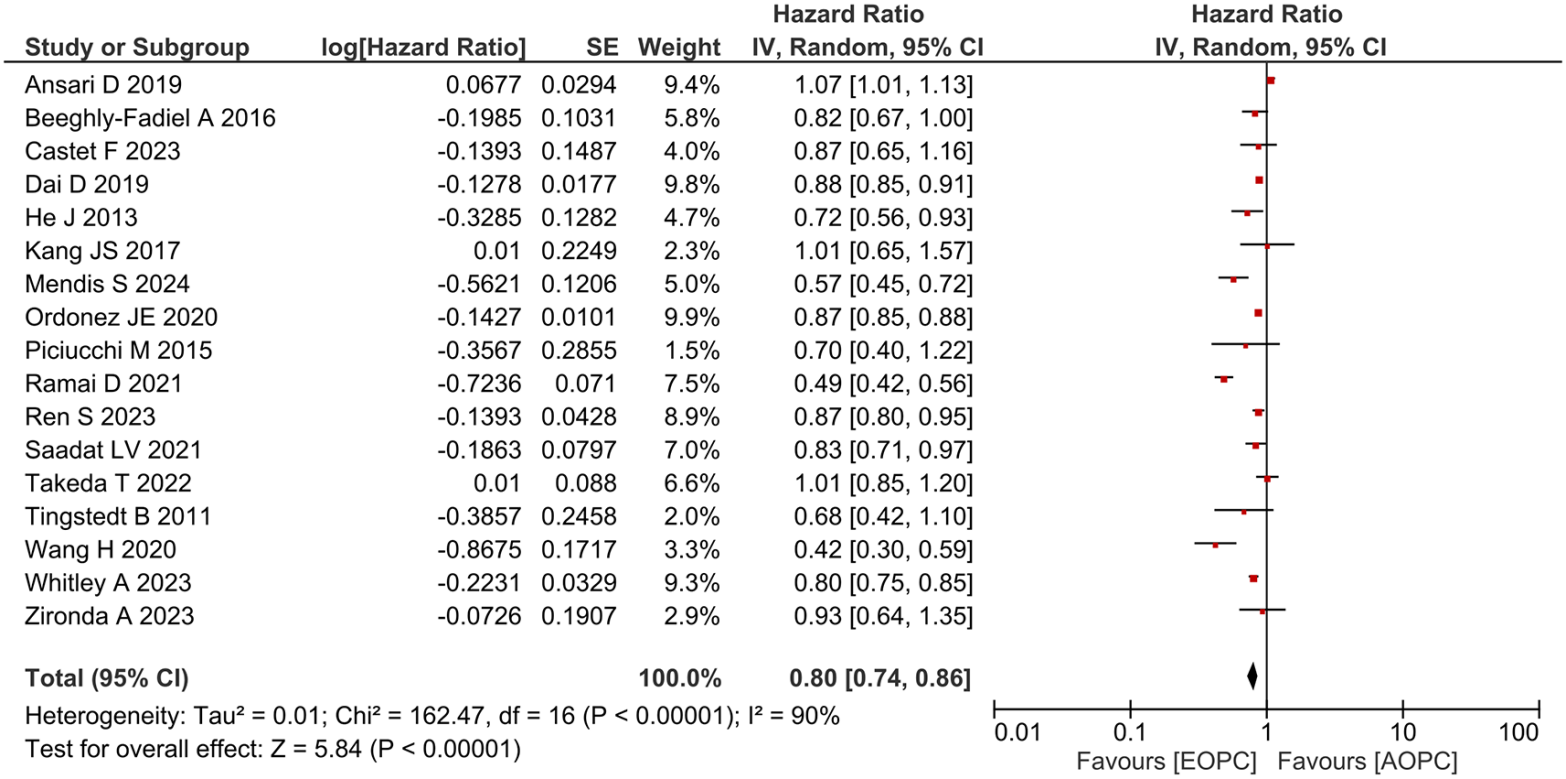
Forest plot of overall survival analysis between EOPC and AOPC patients.

Figure 2 shows the forest plot of the OS analysis of the studies included in the present meta-analysis. The patients with EOPC had a better OS than those with AOPC (aHR = 0.80; 95% confidence interval [CI], 0.74–0.86; *P* < 0.001). The range of median survival for EOPC subjects was 5.7─36.0 months, while the range of median survival for AOPC patients was 4.0─32.0 months.

The sensitivity analysis is shown in Table 3.

**Table 3.** Sensitivity analysis for the pooled overall survival of included studies.

Sensitivity analysis	No. of studies	Pooled Adjusted Hazard Ratio (95% CI)
Only published adjusted hazard ratio	7	0.80 (95% CI 0.68─0.96, P = 0.01)
Only studies with ‘overall survival’ as the primary outcome	15	0.81 (95% CI 0.73─0.89, P < 0.001)
<50 years old (EOPC) cut off	12	0.86 (95% CI 0.79─0.93, P = 0.0003)
<45 years old (EOPC) cut off	3	0.88 (95% CI 0.85─0.91, P < 0.001)
<40 years old (EOPC) cut off	2	0.47 (95% CI 0.42─0.54, P < 0.001)
<50 years old (EOPC) vs. >50 years old (AOPC) only	11	0.86 (95% CI 0.79─0.93, P = 0.0003)
<45 years old (EOPC) vs. >45 years old (AOPC) only	2	0.88 (95% CI 0.85─0.91, P < 0.001)
Adjusted for treatment received	8	0.82 (95% CI 0.74─0.92, P < 0.001)
Adjusted for cancer stage	12	0.80 (95% CI 0.73─0.87, P < 0.001)
Adjusted for comorbidities	1	0.87 (95% CI 0.85─0.88, P < 0.001)
Studies with propensity-score based method	4	0.95 (95% CI 0.81─1.12, P = 0.56)
Studies with Cox regression method	9	0.79 (95% CI 0.70─0.88, P < 0.001)

The funnel plot for OS analysis is shown in Figure S1. Egger’s test showed no significant publication bias ( *P* = 0.227).

We also performed a pooled analysis of studies that included other types of survival analyses. Pooled CSS analysis (n = 4), as seen in Figure S2, showed that patients with EOPC had a better CSS than those with AOPC (HR = 0.85; 95% CI, 0.72–1.00; *P* = 0.05). Pooled RFS analysis (n = 4), as seen in Figure S3, showed that patients with EOPC had a similar RFS to those with AOPC (HR = 1.10; 95% CI, 0.78–1.54; *P* = 0.60). The pooled PFS (n = 3), as seen in Figure S4, also showed that patients with EOPC had a similar PFS to those with AOPC (HR = 0.84; 95% CI, 0.61–1.17; *P* = 0.30). Only one study reported DFS, which showed that patients with EOPC had a worse DFS than those with AOPC (HR = 2.40; 95% CI, 1.13–5.10; *P* = 0.02). ^32^

**Overall survival in patients undergoing surgery**

**Figure 3.**
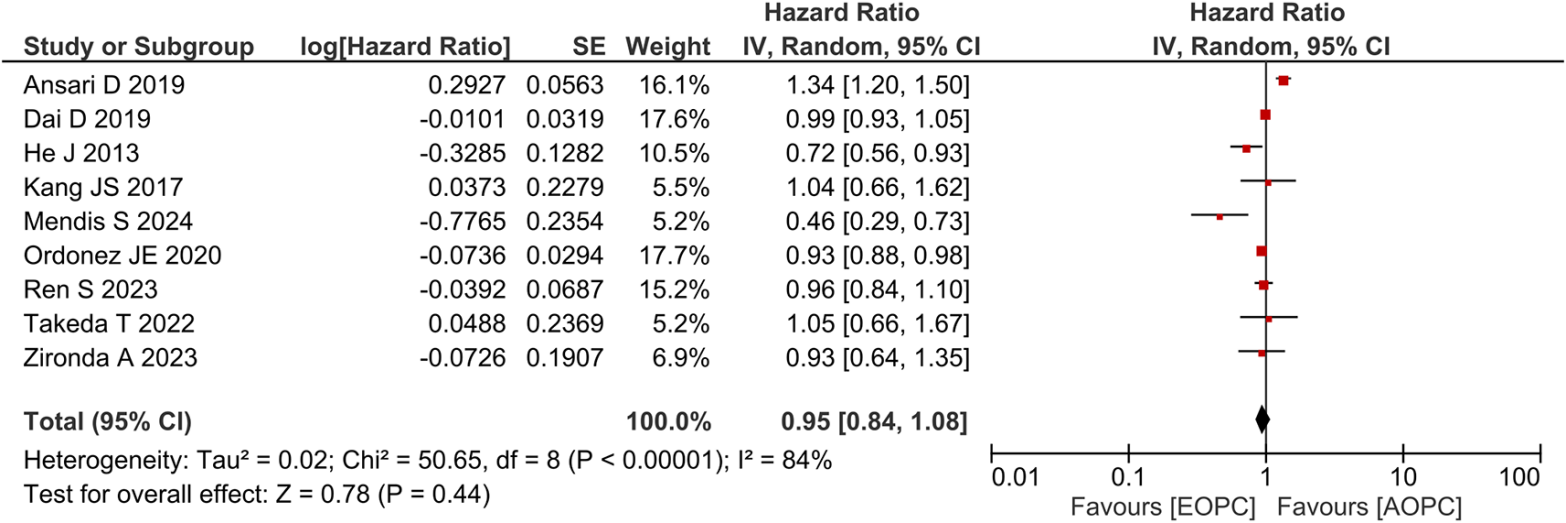
Forest plot of subgroup overall survival analysis between EOPC and AOPC patients who received surgery.

Figure 3 shows the forest plot for studies that performed subgroup OS analyses in patients undergoing surgery (n = 9), the result of which showed that patients with EOPC who underwent surgery had a similar OS to those with AOPC who underwent surgery (aHR = 0.95; 95% CI, 0.84–1.08; *P* = 0.44).

### Distant metastasis

Figure S5 shows the pooled analysis of the risk ratio (RR) of distant metastasis between patients with EOPC and those with AOPC. Twelve studies were included in the pooled analysis, the results of which showed that patients with EOPC had an increased risk of distant metastasis (stage IV) than those with AOPC (RR = 1.08; 95% CI, 1.03–1.13; *P* = 0.001).

### Treatments received

**Surgery**

Figure S6 shows the pooled analysis of the RR of the rate of surgery between patients with EOPC and those with AOPC, the results of which showed that patients with EOPC underwent more surgeries than those with AOPC (RR = 1.22; 95% CI, 1.13–1.32; *P* < 0.001).

**Chemotherapy**

Figure S7 shows the pooled analysis of the RR of the rate of chemotherapy between patients with EOPC and those with AOPC, the results of which showed that patients with EOPC received more chemotherapy than those with AOPC (RR = 1.31; 95% CI, 1.25–1.37; *P* < 0.001).

**Radiotherapy**

Figure S8 shows a pooled analysis of the RR of the rate of radiotherapy between patients with EOPC and those with AOPC, the results of which showed that patients with EOPC underwent more radiotherapy than those with AOPC (RR = 1.35; 95% CI, 1.32–1.38; *P* < 0.001).

## Discussion

The relationship between age at diagnosis and the survival of patients with cancer is complex. Some studies have shown that early-onset breast, lung, central nervous system, and soft tissue cancers are associated with poor prognosis. ^33^^,^^34^ Other studies, however, have shown that patients with early-onset cancer had a better OS than those with late-onset cancer, such as colorectal cancer. ^35^ The results of our meta-analysis fall in line with the latter, showing that EOPC patients had better prognosis than AOPC patients. Our findings are in line with the results of most previous studies. For example, Beeghly-Fadiel et al. ^9^ showed that patients with EOPC had a better OS than those with AOPC, independent of other factors. They also showed that the mortality rate increased significantly after the age of 60 years in patients with EOPC. A study by Ordonez et al. ^14^ showed similar results. Although patients with EOPC presented with several risk factors that are typically associated with worse survival (e.g., more advanced stage, male sex, and non-caput tumor), they still had a better OS than patients with AOPC.

Several other studies, however, have reported contradictory results. Ansari et al. ^4^ analyzed 72,906 patients with PDAC from the SEER registry, and after propensity score matching, found that patients with EOPC had a shorter CSS than those with AOPC. This result was true even after controlling for other factors, such as cancer stage and treatment received by the patients. They also found that patients with EOPC were also more often diagnosed at more advanced AJCC stages and received more treatments (surgery, radiotherapy, and chemotherapy) than patients with AOPC. Another registry-based study in Japan also found that younger patients had worse survival rates than older patients. Similarly, the aforementioned study showed that younger patients were often diagnosed at more advanced stages than older patients; however, they found that younger patients underwent fewer surgeries and achieved fewer R0 resections than older patients. When subgroup analysis of resected patients was performed, there was no difference in the survival rates between younger and older patients. ^15^ The aforementioned study was not included in the pooled analysis, however, because the survival analysis was not adjusted for cancer stage.

The pooled analysis in the present study also showed that patients with EOPC had a higher rate of distant metastasis than those with AOPC, a phenomenon which was also observed in previous studies. For example, Tingstedt et al. ^7^ found a higher proportion of distant metastasis in patients with EOPC than in those with AOPC. Eguchi et al. ^15^ found that patients with EOPC had a larger tumor size, liver metastasis, and peritonitis carcinomatosa than those with AOPC. It is still unclear why patients with EOPC are more often diagnosed at an advanced cancer stage compared to patients with AOPC. One potential explanation for this might be the underdiagnosis of cancer in younger patients, as clinicians may be less likely to diagnose rare pathologies in younger patients, particularly in the early stages of the disease. Additionally, younger patients are more likely to present to the hospital at a later stage of the disease, due to a reluctance to seek care early. ^36^ Some studies have hypothesized that patients with EOPC may have a more aggressive tumor phenotype than patients with AOPC due to differences in their molecular profiles. ^37^^,^^38^

Several studies have compared the molecular profiles of EOPC and AOPC, with mixed results. Bergmann et al. ^37^ investigated the molecular characteristics of 7 patients with PDAC aged ≤ 40 years old, and found that all of the patients exhibited *SMAD4* inactivation, which was associated with more aggressive tumors. Surprisingly, they also found that most patients had wild-type *KRAS*, which is unusual, as *KRAS* mutations are commonly found in patients with PDAC (90%). ^16^ Wild-type *KRAS* was also associated with other targetable alterations, such as mismatch repair deficiency. ^38^ In a recent preprint, Ogobuiro et al. ^39^ showed that patients with EOPC with wild-type *KRAS* tumors had fewer *TP53* mutations. Instead, carcinogenesis in EOPC is more likely driven by *NRG1* and *MET* fusions. *BRAF* fusion was observed only in patients with AOPC with wild-type *KRAS.* In a subgroup analysis of patients with wild-type *KRAS*, the patients with EOPC had a better prognosis than those with AOPC; however, there was no difference in the survival of any patients with mutant *KRAS.* These molecular characteristics might explain the different results of prognostic studies comparing patients with EOPC and those with AOPC. Other studies have also shown a higher rate of mutations in several genes in patients with EOPC compared to patients with AOPC, such as *CDKN2A*, *FOXC2*, and *PI3KCA.* ^40^^,^^41^

Whether younger patients had a higher prevalence of pathogenic germline variants (PGVs) than older patients remains unclear. Bannon et al. ^42^ found that patients with EOPC had a higher prevalence of PGVs (most commonly *BRCA1/2* and *MMR*) than patients with AOPC, which was especially true for patients < 42 years old (OR = 4.17; 95% CI, 1.42–11.84; *P*= 0.011). Castet et al. ^19^ found that 22% of patients from the EOPC group and 13% from the AOPC group had PGVs, the most common of which was *BRCA2.* However, *TP53*, *PMS2*, and *MSH6* PGVs were only found in the EOPC group. Additionally, patients with PGVs had a better OS than those without PGVs, independent of other factors. In contrast, Raffene et al. ^43^ found no significant molecular profile differences between the EOPC and AOPC. It is possible that only a certain subset of EOPC patients have distinct molecular profiles than AOPC patients. Intra-tumoral (variability across individual cell populations within a biopsy site) and inter-tumoral heterogeneity (variability across individual cell populations between the primary and the metastatic site) may also be present, which are important confounders in genomic studies. ^41^

Despite showing that patients with EOPC had a higher rate of distant metastasis, the results of the present meta-analysis also showed that patients with EOPC received more treatments than those with AOPC, which might explain why patients with EOPC had longer survival times than those with AOPC, even though they were more often diagnosed at a more advanced stage. This hypothesis was corroborated by a subgroup analysis of patients who underwent surgery, the results of which showed no significant difference in survival between the two groups. This phenomenon has been universally observed in other studies. Saadat et al. ^6^ studied the differences in treatment utilization patterns between patients with EOPC and those with AOPC in the United States. They found that overall, patients with EOPC received more multimodal treatment regimens than those with AOPC, regardless of the cancer stage; therefore, they hypothesized that younger patients would be more willing to seek care, more likely to have private health insurance, have better access to tertiary healthcare centers, and be more fit to undergo treatment. Clinicians were also more willing to prescribe intensive treatments to younger patients because of their longer life expectancies compared to older patients; however, a high percentage of patients with EOPC and AOPC (19% and 39%, respectively) did not receive any treatment. Those who received no treatment tended to be non-White females with no private health insurance, less income, and lower levels of education, suggesting the vital role of the social determinants in the health of patients with PDAC. It is also important to note that most of these studies were conducted in developed countries, whereas patients with EOPC in developing countries may face more barriers to treatment, primarily due to financial hurdles. Younger patients with cancer may have no or inadequate health insurance coverage, limited financial assets, and significant work interruptions, leading to high financial strain. ^44^ Therefore, patients with EOPC in developing countries may have different treatment utilization patterns than those in developed countries.

The present study has several limitations. First, we only included studies written in English, which may have increased publication bias. Second, there was also substantial heterogeneity between the included studies, possibly due to differences in the age cutoffs for EOPC, study time frames that might have lead to different treatment protocols, and the inclusion of covariates in the survival analysis. Therefore, we performed several sensitivity analyses that yielded similar conclusions. Third, the retrospective design of the included studies also means that some data, such as the specific chemotherapeutic agents used and genetic data, may be difficult to obtain. The present study does, however, have several strengths. First, to the best of the authors’ knowledge, this is the first meta-analysis to compare the survival of patients with EOPC to those with AOPC. Finally, we used multiple statistical methods ^21^^,^^27^ to estimate the aHR of several studies to maximize study inclusion and minimize publication bias.

### Ethics and consent

Ethical approval and consent were not required.

## Data Availability

No data associated with this article. Figshare: Supplementary Tables and Figures. https://doi.org/10.6084/m9.figshare.26130982 ^45^ Data is available under the terms of the Creative Commons Attribution 4.0 International license (CC-BY 4.0). Figshare: PRISMA checklist and flowchart for ‘The Association between Early-Onset Pancreatic Ductal Adenocarcinoma and Patient’s Survival: A Systematic Review and Meta-Analysis’. https://doi.org/10.6084/m9.figshare.26548492 ^46^ Data is available under the terms of the Creative Commons Attribution 4.0 International license (CC-BY 4.0).
