# Supplementary material for: The Association between Early-Onset Pancreatic Ductal Adenocarcinoma and Patients Survival: A Systematic Review and Meta-Analysis
Source: F1000Res. 2024 Aug 28;13:976. [Version 1] doi: 10.12688/f1000research.153743.1 (PMC11443187; doi:10.12688/f1000research.153743.1)
Supplement: Peer review history

# Reviewer report: Reviewer response for version 1

**DOI:** 10.5256/f1000research.168677.r322276

**Published:** 2024-09-30

**Authors:** Stefania Bunduc

**Affiliations:** 1 Carol Davila University of Medicine and Pharmacy, Bucharest, Romania; 2 Centre for Translational Medicine, Semmelweis University, Budapest, Hungary

The authors have performed a systematic review and meta analysis evaluating the prognosis differences between early (<= 50 years of age) and average (> 50 years old) onset pancreatic cancer patients.

 The methodology is sound, reporting is appropriate the idea is new and the findings are relevant while the manuscript is well balanced and very well written. The authors may consider the following minor comments:

 Methods section:

 The revised PRISMA statement was published in 2020 - although changes are not major the authors may consider reporting based on it

PRISMA 2020 statement — PRISMA statement (prisma-statement.org)

 - was the protocol previously published on PROSPERO? - if yes, please provide CRD

 - the statistical analysis subsection should be dedicated to explaining how the calculations were performed while the evaluated outcomes - primary and secondary should be reported in a separate subsection 

 Results:

 - tables and figures should stand alone abbreviations should be defined in footnotes

Are the rationale for, and objectives of, the Systematic Review clearly stated?

Yes

Is the statistical analysis and its interpretation appropriate?

I cannot comment. A qualified statistician is required.

If this is a Living Systematic Review, is the ‘living’ method appropriate and is the search schedule clearly defined and justified? (‘Living Systematic Review’ or a variation of this term should be included in the title.)

Not applicable

Are sufficient details of the methods and analysis provided to allow replication by others?

Yes

Are the conclusions drawn adequately supported by the results presented in the review?

Yes

Reviewer Expertise:

gastroenterology; pancreatology; pancreatic cancer; liquid biopsy; meta-analysis

I confirm that I have read this submission and believe that I have an appropriate level of expertise to confirm that it is of an acceptable scientific standard.

# Reviewer report: Reviewer response for version 1

**DOI:** 10.5256/f1000research.168677.r318420

**Published:** 2024-09-11

**Authors:** Yan Wisnu Prajoko

**Affiliations:** 1 Department of Surgical Oncology, Diponegoro University, Semarang, Indonesia

1. The topic of this article is quite interesting and updated. because reviewing the prevalence of early onset pancreatic cancer which has increased quite a bit recently, both in developing countries and especially in developed countries.

 2. The literature review is also quite complete, but the author himself realizes that there are shortcomings, because it only includes articles written in English. 

 3. The review method has also been carried out well and systematically

 4. The results are well written and in fairly good English

 5. Even though the results obtained do not provide much clarity, this study can provide additional knowledge about this scientific field or disease.

Are the rationale for, and objectives of, the Systematic Review clearly stated?

Yes

Is the statistical analysis and its interpretation appropriate?

Yes

If this is a Living Systematic Review, is the ‘living’ method appropriate and is the search schedule clearly defined and justified? (‘Living Systematic Review’ or a variation of this term should be included in the title.)

Not applicable

Are sufficient details of the methods and analysis provided to allow replication by others?

Yes

Are the conclusions drawn adequately supported by the results presented in the review?

Yes

Reviewer Expertise:

surgical oncology

I confirm that I have read this submission and believe that I have an appropriate level of expertise to confirm that it is of an acceptable scientific standard.

# Response

**Published:** 2024-09-12

**Authors:** Kaka Renaldi

**Affiliations:** Universitas Indonesia, Depok, West Java, Indonesia

Thank you for the kind comment and for taking time to review our paper.
